# Dibenzo-18-crown-6–picric acid–water (1/2/3)

**DOI:** 10.1107/S1600536808018485

**Published:** 2008-06-21

**Authors:** Muhammad Idiris Saleh, Eny Kusrini, Mohd Mustaqim Rosli, Hoong-Kun Fun

**Affiliations:** a School of Chemical Sciences, Universiti Sains Malaysia, 11800 USM, Penang, Malaysia; b X-ray Crystallography Unit, School of Physics, Universiti Sains Malaysia, 11800 USM, Penang, Malaysia

## Abstract

In the crown ether ring of the title compound, C_20_H_24_O_6_·2C_6_H_3_N_3_O_7_·3H_2_O, the O—C(H_2_)—C(H_2_)—O torsion angles indicate a *gauche* conformation of the ethyl­eneoxy units, while the C—O—C—C torsion angles indicate planarity of these segments; the dihedral angle between the two benzene rings is 44.53 (13)°. In both picric acid mol­ecules, one of the nitro groups is twisted away from the attached ring. The mol­ecules are linked into chains along the *b* axis *via* inter­molecular O—H⋯O hydrogen bonds. In addition, the crystal structure is stabilized by C—H⋯O hydrogen bonds and π–π inter­actions [centroid–centroid distance between benzene rings = 3.5697 (16) Å].

## Related literature

For bond-length data, see: Allen *et al.* (1987 ▶). For related literature, see: Bush & Truter (1971 ▶); Colquhoun *et al.* (1986 ▶); Kanters *et al.* (1986 ▶); Lu *et al.* (1993*a* ▶,*b* ▶); Robinson *et al.* (1987 ▶); Saleh *et al.* (1996 ▶, 1997 ▶); You *et al.* (2002 ▶); Zhou *et al.* (1996 ▶).
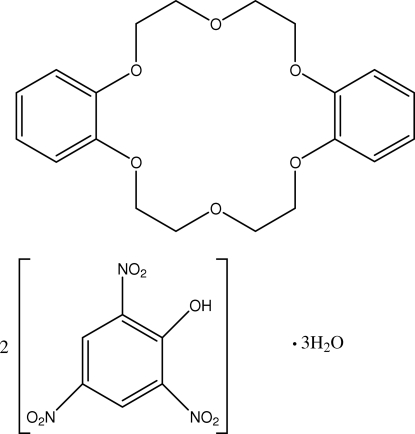


## Experimental

### 

#### Crystal data

C_20_H_24_O_6_·2C_6_H_3_N_3_O_7_·3H_2_O
*M* *_r_* = 872.67
Orthorhombic, 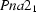

*a* = 16.4192 (2) Å
*b* = 7.0845 (1) Å
*c* = 31.4135 (4) Å
*V* = 3654.08 (8) Å^3^
*Z* = 4
Mo *K*α radiation
μ = 0.14 mm^−1^
*T* = 100.0 (1) K
0.36 × 0.32 × 0.16 mm

#### Data collection

Bruker SMART APEXII CCD area-detector diffractometer
Absorption correction: multi-scan (*SADABS*; Bruker, 2005 ▶) *T*_min_ = 0.952, *T*_max_ = 0.979
37248 measured reflections
5431 independent reflections
4559 reflections with *I* > 2σ(*I*)
*R*_int_ = 0.045

#### Refinement

*R*[*F* ^2^ > 2σ(*F* ^2^)] = 0.044
*wR*(*F* ^2^) = 0.110
*S* = 1.06
5431 reflections
550 parameters
1 restraint
H-atom parameters constrained
Δρ_max_ = 0.40 e Å^−3^
Δρ_min_ = −0.33 e Å^−3^

### 

Data collection: *APEX2* (Bruker, 2005 ▶); cell refinement: *APEX2*; data reduction: *SAINT* (Bruker, 2005 ▶); program(s) used to solve structure: *SHELXTL* (Sheldrick, 2008 ▶); program(s) used to refine structure: *SHELXTL*; molecular graphics: *SHELXTL*; software used to prepare material for publication: *SHELXTL* and *PLATON* (Spek, 2003 ▶).

## Supplementary Material

Crystal structure: contains datablocks global, I. DOI: 10.1107/S1600536808018485/ci2617sup1.cif

Structure factors: contains datablocks I. DOI: 10.1107/S1600536808018485/ci2617Isup2.hkl

Additional supplementary materials:  crystallographic information; 3D view; checkCIF report

## Figures and Tables

**Table 1:** Hydrogen-bond geometry (Å, °)

*D*—H⋯*A*	*D*—H	H⋯*A*	*D*⋯*A*	*D*—H⋯*A*
O7—H1*O*7⋯O3*W*	1.00	1.92	2.618 (3)	124
O7—H1*O*7⋯O13	1.00	1.84	2.664 (3)	138
O7—H1*O*7⋯N3	1.00	2.49	2.985 (3)	110
O1*W*—H1*W*1⋯O6	0.87	2.14	2.978 (3)	162
O1*W*—H2*W*1⋯O2*W*	0.94	2.03	2.900 (4)	152
O2*W*—H1*W*2⋯O1^i^	0.85	2.56	3.215 (3)	135
O2*W*—H1*W*2⋯O2^i^	0.85	2.45	3.265 (3)	162
O2*W*—H2*W*2⋯O4^i^	0.85	2.42	3.198 (3)	152
O2*W*—H2*W*2⋯O5^i^	0.85	2.43	3.155 (3)	144
O3*W*—H1*W*3⋯O3	0.85	2.02	2.861 (3)	173
O3*W*—H2*W*3⋯O1*W*	0.95	1.96	2.881 (3)	163
O14—H14*B*⋯O1*W*	0.78	2.06	2.732 (3)	144
O14—H14*B*⋯O20	0.78	2.05	2.632 (3)	131
C3—H3*A*⋯O11^ii^	0.93	2.60	3.323 (4)	136
C7—H7*A*⋯O6^iii^	0.97	2.58	3.393 (3)	142
C7—H7*B*⋯O19^iv^	0.97	2.59	3.135 (4)	116
C9—H9*A*⋯O12^iii^	0.97	2.39	3.341 (4)	165
C19—H19*A*⋯O19^v^	0.97	2.53	3.301 (3)	137

Symmetry codes: (i) 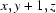; (ii) 
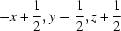
; (iii) 
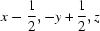
; (iv) 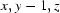; (v) 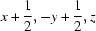.

**Fig. 1.**
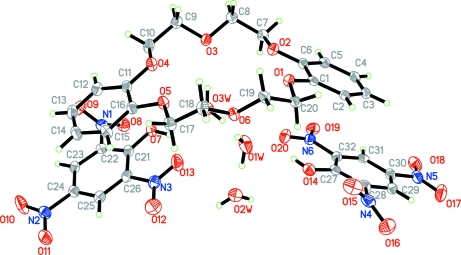
The asymmetric unit of the title compound, showing 50% probability displacement ellipsoids and the atomic numbering.

**Fig. 2.**
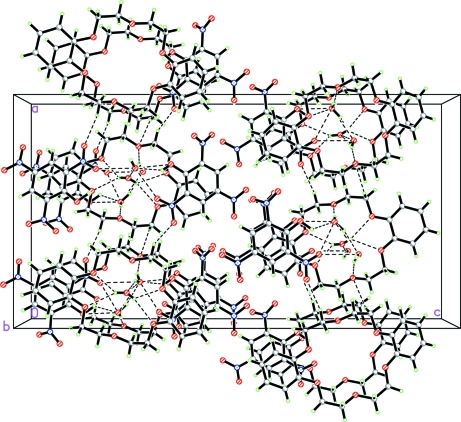
The crystal packing of the title compound, viewed down the b axis. Hydrogen bonds are shown as dashed lines.

## supplementary crystallographic information

### Comment

The lanthanide complexes with crown ethers have been previously reported e.g. [Ln(NO_3_)_2_(H_2_O)_2_(DB30C10)_2_].[Ln(NO_3_)_5_]. CH_3_CN {Ln = Sm - Lu} (Lu *et al.*,  1993*a*), [Gd(NO_3_)_3_(H_2_O)_3_].(DB24C8) (Lu *et al.*,  1993*b*) and Ln(Pic)_3_.(B15C5)_2_nH_2_O {Ln = Nd, Sm, Er} (Zhou *et al.*,  1996) where DB30C10 is dibenzo-30-crown-10, DB24C8 is dibenzo-24-crown-8, B15C5 is benzo-15-crown-5 and Pic is picrate anion. The complexation of dibenzo-18-crown-6 (DB18C6) with alkali and transition metal ions have also been reported e.g. NaBr(DB18C6).2H_2_O (Bush & Truter, 1971), [Ga(CH_3_)_3_]_2_(DB18C6) (Robinson *et al.*, 1987) and [(H_3_O(DB18C6)]_2_[HPMo_12_O_40_].DB18C6.3CH_3_CN.H_2_O (You *et al.*, 2002) where HPMo_12_O_40_ is 12-molybdophosphate acid. Additionally, different products without lanthanide coordination with crown ether in the presence of picric acid have also been observed namely [NH_4_(Pic)(DB18C6)] (Kanters *et al.*,  1986), DB18C6.2HPic and DB24C8.2HPic (Colquhoun *et al.*,  1986), D15C5.2HPic (Saleh *et al.*,  1996) where DD18C6 is *N,N'*-dibenzyl-1,14, 10,13,-tetraoxa-7,16-diazacyclooctadecane.

In our study, no complexation product was obtained from a solution mixture containing dibenzo-18-crown-6, terbium nitrate and picric acid and instead the formation of DB18C6 with three water molecules in the presence of two picric acid have taken place. The product has a red colour consistent with a charge transfer interaction between the π-electron-rich benzene rings of the DB18C6 and the lack of π-electron in the HPic molecules.

The molecular structure of the title compound is shown in Fig.1. Bond lengths and angles have normal values (Allen *et al.*,  1987). The dihedral angle between the two benzene rings (C1-C6 and C11-C16) in dibenzo-18-crown-6 unit is 44.53 (13)°. In the crown ether, the O-C(H_2_)-C(H_2_)-O torsion angles indicate a gauche conformation of the ethyleneoxy units, while the C-O-C-C torsion angles indicate planarity of these segments. In both picric acid units, one of the nitro groups is twisted away from the attached ring [O8—N1—C22—C21 = 52.9 (4)°, O9—N1—C22—C23 49.9 (4)°, O15—N4—C28—C29 = 146.4 (3)° and O16—N4—C28—C27 = 148.9 (3)°].

In the crystal structure, O—H···O, O—H···N and C—H···O hydrogen bonds are observed  (Table 1). The molecules are linked into chains along the *b* axis via intermolecular O—H···O hydrogen bonds (Fig. 2). In addition, π-π interactions involving the C1-C6 (centroid Cg1) and C27-C32 (centroid Cg2) benzene rings are observed, with a Cg1···Cg2(x, -1+y, z) distance of 3.5697 (16) Å.

### Experimental

The title compound was prepared by the reaction of dibenzo-18-crown-6 (0.17 g, 0.46 mmol) and terbium nitrate (0.43 g, 1 mmol) in the presence of picric acid (0.93 g, 4.06 mmol) in a CH_3_CN-CH_3_OH-CHCl_3_-H_2_O (2:1:1:1 v/v) solution (20 ml). The solution was heated in a water bath with continous stirring for 5 min at 313-323 K. The solution was left to evaporate at room temperature. Red crystals were obtained after one week (yield 90%, decomposition point 381.6-412.0 K). Elemental analysis data: Calcuclated (found): C 44.00 (45.87), H 4.13 (4.32), N 9.63 (8.38)%.

### Refinement

O-bound H atoms were located initially in a difference Fourier map and then constrained to ride on the parent O atom. C-bound H atoms were positioned geometrically and refined using a riding model with C-H = 0.93-0.97 Å. All H atoms were refined with *U*_iso_(H) = 1.5*U*_eq_(O) and 1.2*U*_eq_(C). In the absence of significant anomalous scattering effects, Friedel pairs were averaged.

### Crystal data

**Table:**

C_20_H_24_O_6_·2C_6_H_3_N_3_O_7_·3H_2_O	*F*_000_ = 1816
*M**_r_* = 872.67	*D*_x_ = 1.586 Mg m^−^^3^
Orthorhombic, *P**n**a*2_1_	Mo *K*α radiation λ = 0.71073 Å
Hall symbol: P 2c -2n	Cell parameters from 8814 reflections
*a* = 16.4192 (2) Å	θ = 2.6–30.1º
*b* = 7.0845 (1) Å	µ = 0.14 mm^−^^1^
*c* = 31.4135 (4) Å	*T* = 100.0 (1) K
*V* = 3654.08 (8) Å^3^	Block, red
*Z* = 4	0.36 × 0.32 × 0.16 mm

### Data collection

**Table:**

Bruker SMART APEXII CCD area-detector diffractometer	5431 independent reflections
Radiation source: fine-focus sealed tube	4559 reflections with *I* > 2σ(*I*)
Monochromator: graphite	*R*_int_ = 0.045
Detector resolution: 8.33 pixels mm^-1^	θ_max_ = 30.1º
*T* = 100.0(1) K	θ_min_ = 2.5º
ω scans	*h* = −17→23
Absorption correction: multi-scan(SADABS; Bruker, 2005)	*k* = −9→9
*T*_min_ = 0.952, *T*_max_ = 0.979	*l* = −43→44
37248 measured reflections	

### Refinement

**Table:**

Refinement on *F*^2^	Secondary atom site location: difference Fourier map
Least-squares matrix: full	Hydrogen site location: inferred from neighbouring sites
*R*[*F*^2^ > 2σ(*F*^2^)] = 0.044	H-atom parameters constrained
*wR*(*F*^2^) = 0.110	*w* = 1/[σ^2^(*F*_o_^2^) + (0.0586*P*)^2^ + 0.3893*P*] where *P* = (*F*_o_^2^ + 2*F*_c_^2^)/3
*S* = 1.06	(Δ/σ)_max_ = 0.001
5431 reflections	Δρ_max_ = 0.40 e Å^−^^3^
550 parameters	Δρ_min_ = −0.33 e Å^−^^3^
1 restraint	Extinction correction: none
Primary atom site location: structure-invariant direct methods	

### Special details

**Table:**

Experimental. The low-temperature data was collected with the Oxford Cyrosystem Cobra low-temperature attachment.
Geometry. All esds (except the esd in the dihedral angle between two l.s. planes) are estimated using the full covariance matrix. The cell esds are taken into account individually in the estimation of esds in distances, angles and torsion angles; correlations between esds in cell parameters are only used when they are defined by crystal symmetry. An approximate (isotropic) treatment of cell esds is used for estimating esds involving l.s. planes.
Refinement. Refinement of F^2^ against ALL reflections. The weighted R-factor wR and goodness of fit S are based on F^2^, conventional R-factors R are based on F, with F set to zero for negative F^2^. The threshold expression of F^2^ > 2sigma(F^2^) is used only for calculating R-factors(gt) etc. and is not relevant to the choice of reflections for refinement. R-factors based on F^2^ are statistically about twice as large as those based on F, and R- factors based on ALL data will be even larger.

### Fractional atomic coordinates and isotropic or equivalent isotropic displacement parameters (Å^2^)

**Table:**

	*x*	*y*	*z*	*U*_iso_*/*U*_eq_	
O1	0.17228 (11)	0.0965 (3)	0.33490 (6)	0.0194 (4)	
O2	0.02175 (11)	0.0646 (3)	0.31441 (6)	0.0205 (4)	
O3	−0.03139 (11)	0.0282 (3)	0.22594 (6)	0.0225 (4)	
O4	0.09157 (11)	0.0249 (3)	0.16058 (6)	0.0239 (4)	
O5	0.23885 (11)	0.0985 (3)	0.18147 (6)	0.0226 (4)	
O6	0.29382 (11)	0.1294 (3)	0.26846 (6)	0.0193 (4)	
C1	0.11872 (16)	0.1717 (4)	0.36375 (8)	0.0179 (5)	
C2	0.13993 (16)	0.2525 (4)	0.40216 (8)	0.0197 (5)	
H2A	0.1944	0.2613	0.4099	0.024*	
C3	0.07934 (18)	0.3210 (4)	0.42944 (8)	0.0220 (5)	
H3A	0.0936	0.3756	0.4553	0.026*	
C4	−0.00162 (18)	0.3077 (4)	0.41795 (9)	0.0233 (6)	
H4A	−0.0418	0.3546	0.4360	0.028*	
C5	−0.02333 (17)	0.2239 (4)	0.37928 (8)	0.0208 (5)	
H5A	−0.0780	0.2141	0.3718	0.025*	
C6	0.03588 (16)	0.1556 (4)	0.35220 (8)	0.0194 (5)	
C7	−0.06112 (16)	0.0602 (4)	0.30033 (9)	0.0219 (5)	
H7A	−0.0794	0.1867	0.2934	0.026*	
H7B	−0.0958	0.0107	0.3227	0.026*	
C8	−0.06605 (16)	−0.0636 (4)	0.26173 (8)	0.0230 (6)	
H8A	−0.0373	−0.1808	0.2671	0.028*	
H8B	−0.1226	−0.0938	0.2559	0.028*	
C9	−0.02913 (17)	−0.0983 (5)	0.19033 (9)	0.0260 (6)	
H9A	−0.0838	−0.1413	0.1838	0.031*	
H9B	0.0038	−0.2077	0.1973	0.031*	
C10	0.00626 (17)	0.0008 (5)	0.15248 (9)	0.0264 (6)	
H10A	−0.0019	−0.0738	0.1269	0.032*	
H10B	−0.0197	0.1225	0.1485	0.032*	
C11	0.13815 (17)	0.1008 (4)	0.12882 (8)	0.0208 (5)	
C12	0.11157 (18)	0.1340 (4)	0.08740 (9)	0.0247 (6)	
H12A	0.0577	0.1105	0.0799	0.030*	
C13	0.16674 (19)	0.2032 (4)	0.05716 (9)	0.0278 (6)	
H13A	0.1496	0.2247	0.0294	0.033*	
C14	0.24573 (19)	0.2392 (4)	0.06832 (9)	0.0277 (6)	
H14A	0.2819	0.2846	0.0480	0.033*	
C15	0.27260 (18)	0.2088 (4)	0.10966 (9)	0.0243 (6)	
H15A	0.3263	0.2346	0.1170	0.029*	
C16	0.21896 (18)	0.1396 (4)	0.14004 (8)	0.0217 (5)	
C17	0.32251 (16)	0.1243 (4)	0.19333 (9)	0.0234 (6)	
H17A	0.3351	0.2577	0.1955	0.028*	
H17B	0.3580	0.0686	0.1721	0.028*	
C18	0.33476 (16)	0.0296 (4)	0.23549 (9)	0.0230 (6)	
H18A	0.3142	−0.0986	0.2341	0.028*	
H18B	0.3925	0.0238	0.2419	0.028*	
C19	0.30305 (16)	0.0303 (4)	0.30759 (8)	0.0201 (5)	
H19A	0.3603	0.0237	0.3150	0.024*	
H19B	0.2829	−0.0977	0.3044	0.024*	
C20	0.25708 (15)	0.1275 (4)	0.34246 (8)	0.0200 (5)	
H20A	0.2726	0.0762	0.3699	0.024*	
H20B	0.2690	0.2616	0.3423	0.024*	
O7	0.04207 (12)	0.5129 (3)	0.14168 (6)	0.0255 (4)	
H1O7	0.0768	0.5063	0.1676	0.038*	
O8	−0.08444 (13)	0.6214 (3)	0.09050 (7)	0.0345 (5)	
O9	−0.05274 (14)	0.4429 (4)	0.03674 (7)	0.0388 (6)	
O10	0.18091 (15)	0.7752 (5)	−0.03288 (7)	0.0483 (7)	
O11	0.28256 (15)	0.8540 (4)	0.00640 (8)	0.0461 (7)	
O12	0.28468 (13)	0.6902 (4)	0.15276 (8)	0.0399 (6)	
O13	0.18305 (14)	0.5543 (4)	0.18259 (7)	0.0398 (6)	
N1	−0.03549 (14)	0.5511 (4)	0.06579 (8)	0.0251 (5)	
N2	0.21475 (14)	0.7882 (4)	0.00136 (8)	0.0273 (5)	
N3	0.21571 (15)	0.6291 (3)	0.15208 (8)	0.0264 (5)	
C21	0.08740 (17)	0.5840 (4)	0.11077 (8)	0.0219 (5)	
C22	0.05087 (16)	0.6039 (4)	0.07059 (9)	0.0212 (5)	
C23	0.09073 (16)	0.6653 (4)	0.03478 (9)	0.0215 (5)	
H23A	0.0646	0.6701	0.0085	0.026*	
C24	0.17102 (16)	0.7197 (4)	0.03915 (9)	0.0229 (5)	
C25	0.21134 (16)	0.7102 (4)	0.07728 (9)	0.0228 (6)	
H25A	0.2653	0.7486	0.0795	0.027*	
C26	0.16965 (17)	0.6419 (4)	0.11252 (9)	0.0223 (5)	
O14	0.20351 (12)	0.6284 (3)	0.35095 (6)	0.0259 (4)	
H14B	0.1775	0.5883	0.3321	0.039*	
O15	0.32541 (13)	0.5815 (4)	0.40398 (8)	0.0403 (6)	
O16	0.33764 (14)	0.8326 (4)	0.44252 (8)	0.0440 (6)	
O17	0.09655 (17)	1.0026 (4)	0.52185 (7)	0.0429 (6)	
O18	−0.02186 (15)	0.9577 (3)	0.49381 (8)	0.0367 (5)	
O19	−0.04855 (12)	0.6963 (3)	0.35674 (7)	0.0290 (5)	
O20	0.05487 (13)	0.5990 (3)	0.32031 (6)	0.0302 (5)	
N4	0.29744 (15)	0.7191 (4)	0.42194 (8)	0.0304 (6)	
N5	0.05197 (17)	0.9451 (4)	0.49329 (8)	0.0299 (6)	
N6	0.02454 (15)	0.6709 (3)	0.35237 (8)	0.0241 (5)	
C27	0.16382 (16)	0.6979 (4)	0.38383 (8)	0.0195 (5)	
C28	0.20853 (16)	0.7523 (4)	0.42021 (9)	0.0222 (5)	
C29	0.17357 (18)	0.8335 (4)	0.45523 (9)	0.0245 (6)	
H29A	0.2055	0.8720	0.4781	0.029*	
C30	0.09002 (17)	0.8576 (4)	0.45611 (8)	0.0216 (5)	
C31	0.04172 (17)	0.8047 (4)	0.42235 (9)	0.0206 (5)	
H31A	−0.0145	0.8201	0.4233	0.025*	
C32	0.07929 (16)	0.7278 (4)	0.38689 (8)	0.0199 (5)	
O1W	0.18431 (15)	0.4638 (4)	0.27295 (7)	0.0412 (6)	
H1W1	0.2163	0.3723	0.2656	0.062*	
H2W1	0.1914	0.5915	0.2655	0.062*	
O2W	0.14735 (14)	0.8507 (3)	0.24985 (8)	0.0364 (5)	
H1W2	0.1246	0.9207	0.2683	0.055*	
H2W2	0.1497	0.9124	0.2267	0.055*	
O3W	0.04390 (14)	0.3916 (3)	0.22039 (7)	0.0332 (5)	
H1W3	0.0251	0.2805	0.2233	0.050*	
H2W3	0.0825	0.4299	0.2411	0.050*	

### Atomic displacement parameters (Å^2^)

**Table:**

	*U*^11^	*U*^22^	*U*^33^	*U*^12^	*U*^13^	*U*^23^
O1	0.0148 (8)	0.0239 (9)	0.0195 (9)	0.0007 (7)	0.0003 (7)	−0.0020 (7)
O2	0.0156 (9)	0.0249 (10)	0.0211 (9)	0.0016 (7)	−0.0020 (7)	−0.0030 (7)
O3	0.0206 (10)	0.0275 (11)	0.0195 (9)	−0.0027 (8)	0.0004 (7)	−0.0026 (8)
O4	0.0170 (9)	0.0357 (11)	0.0189 (9)	−0.0007 (8)	−0.0011 (7)	0.0010 (8)
O5	0.0174 (9)	0.0322 (11)	0.0183 (9)	−0.0007 (8)	−0.0005 (7)	0.0020 (8)
O6	0.0176 (9)	0.0231 (10)	0.0173 (8)	0.0037 (7)	0.0004 (7)	0.0023 (7)
C1	0.0201 (12)	0.0146 (12)	0.0190 (12)	−0.0006 (9)	0.0024 (10)	0.0041 (9)
C2	0.0212 (12)	0.0183 (13)	0.0197 (12)	−0.0032 (10)	−0.0002 (10)	0.0035 (10)
C3	0.0307 (14)	0.0204 (13)	0.0150 (12)	−0.0013 (11)	0.0008 (10)	0.0015 (10)
C4	0.0299 (14)	0.0211 (14)	0.0190 (12)	0.0018 (11)	0.0064 (10)	0.0028 (10)
C5	0.0196 (12)	0.0214 (13)	0.0214 (12)	0.0009 (10)	0.0032 (10)	0.0027 (10)
C6	0.0218 (12)	0.0187 (13)	0.0177 (11)	0.0009 (10)	0.0007 (10)	0.0019 (10)
C7	0.0137 (12)	0.0288 (15)	0.0231 (13)	−0.0004 (10)	−0.0016 (10)	0.0009 (11)
C8	0.0142 (12)	0.0315 (15)	0.0233 (13)	−0.0036 (10)	−0.0013 (10)	0.0013 (11)
C9	0.0188 (13)	0.0347 (16)	0.0245 (14)	−0.0062 (11)	0.0012 (11)	−0.0065 (12)
C10	0.0214 (13)	0.0370 (16)	0.0206 (13)	−0.0007 (11)	−0.0046 (11)	−0.0049 (12)
C11	0.0239 (13)	0.0201 (13)	0.0183 (12)	0.0025 (10)	0.0023 (10)	−0.0008 (10)
C12	0.0278 (14)	0.0245 (14)	0.0220 (13)	0.0057 (11)	−0.0033 (11)	−0.0010 (11)
C13	0.0384 (17)	0.0267 (15)	0.0183 (13)	0.0075 (12)	0.0002 (11)	−0.0005 (11)
C14	0.0355 (16)	0.0268 (15)	0.0208 (13)	0.0038 (12)	0.0078 (12)	0.0041 (11)
C15	0.0240 (14)	0.0261 (14)	0.0229 (13)	0.0007 (11)	0.0044 (11)	0.0042 (11)
C16	0.0257 (14)	0.0215 (13)	0.0180 (12)	0.0028 (11)	0.0010 (10)	0.0015 (10)
C17	0.0156 (12)	0.0315 (15)	0.0230 (13)	−0.0007 (11)	0.0031 (10)	0.0014 (11)
C18	0.0186 (13)	0.0280 (14)	0.0223 (13)	0.0046 (11)	0.0013 (10)	−0.0012 (11)
C19	0.0160 (11)	0.0225 (13)	0.0218 (12)	0.0021 (10)	−0.0007 (10)	0.0056 (10)
C20	0.0162 (12)	0.0243 (14)	0.0195 (12)	−0.0027 (10)	−0.0010 (10)	0.0030 (10)
O7	0.0242 (10)	0.0329 (11)	0.0195 (9)	0.0007 (8)	0.0015 (7)	0.0023 (8)
O8	0.0232 (11)	0.0472 (14)	0.0329 (11)	−0.0034 (9)	0.0035 (9)	−0.0018 (10)
O9	0.0335 (12)	0.0450 (14)	0.0378 (12)	−0.0024 (10)	−0.0104 (10)	−0.0134 (11)
O10	0.0374 (13)	0.083 (2)	0.0246 (11)	−0.0052 (13)	0.0017 (10)	0.0164 (12)
O11	0.0358 (13)	0.0645 (18)	0.0381 (13)	−0.0178 (12)	0.0138 (10)	−0.0073 (12)
O12	0.0249 (11)	0.0551 (15)	0.0399 (13)	−0.0047 (10)	−0.0115 (10)	0.0029 (11)
O13	0.0362 (13)	0.0625 (17)	0.0207 (10)	−0.0072 (11)	−0.0047 (9)	0.0043 (10)
N1	0.0214 (11)	0.0278 (13)	0.0261 (12)	−0.0022 (9)	−0.0042 (10)	0.0019 (10)
N2	0.0242 (12)	0.0275 (13)	0.0302 (13)	0.0034 (10)	0.0071 (10)	0.0011 (10)
N3	0.0261 (12)	0.0295 (13)	0.0236 (12)	0.0017 (10)	−0.0040 (10)	−0.0014 (10)
C21	0.0237 (14)	0.0226 (13)	0.0195 (12)	0.0026 (10)	0.0016 (10)	−0.0018 (10)
C22	0.0193 (12)	0.0205 (13)	0.0239 (13)	0.0010 (10)	−0.0008 (10)	−0.0009 (11)
C23	0.0248 (13)	0.0216 (13)	0.0182 (12)	0.0050 (11)	−0.0023 (10)	−0.0003 (10)
C24	0.0229 (13)	0.0217 (14)	0.0240 (13)	0.0029 (11)	0.0041 (11)	−0.0001 (11)
C25	0.0188 (12)	0.0240 (14)	0.0256 (13)	0.0037 (10)	−0.0004 (10)	−0.0036 (11)
C26	0.0213 (13)	0.0247 (14)	0.0209 (12)	0.0037 (11)	−0.0030 (10)	−0.0031 (10)
O14	0.0263 (10)	0.0257 (10)	0.0257 (10)	0.0009 (8)	0.0046 (8)	0.0024 (8)
O15	0.0242 (11)	0.0390 (14)	0.0577 (16)	0.0068 (10)	0.0023 (11)	0.0085 (12)
O16	0.0276 (12)	0.0609 (17)	0.0435 (14)	−0.0110 (11)	−0.0089 (10)	−0.0007 (12)
O17	0.0616 (17)	0.0411 (14)	0.0258 (11)	0.0082 (12)	−0.0068 (11)	−0.0077 (10)
O18	0.0403 (13)	0.0372 (13)	0.0326 (11)	0.0081 (10)	0.0125 (10)	0.0039 (10)
O19	0.0199 (10)	0.0311 (11)	0.0360 (11)	−0.0020 (8)	−0.0055 (9)	0.0018 (9)
O20	0.0365 (12)	0.0303 (11)	0.0238 (10)	0.0038 (9)	−0.0043 (9)	−0.0057 (9)
N4	0.0196 (12)	0.0391 (16)	0.0323 (13)	−0.0040 (11)	−0.0009 (10)	0.0122 (12)
N5	0.0426 (16)	0.0243 (13)	0.0228 (12)	0.0038 (11)	0.0046 (11)	0.0027 (10)
N6	0.0280 (12)	0.0209 (12)	0.0233 (11)	−0.0010 (9)	−0.0019 (10)	0.0004 (9)
C27	0.0222 (13)	0.0157 (12)	0.0206 (12)	−0.0003 (10)	0.0023 (10)	0.0034 (9)
C28	0.0196 (12)	0.0232 (14)	0.0239 (13)	−0.0001 (10)	−0.0014 (10)	0.0075 (11)
C29	0.0287 (14)	0.0239 (14)	0.0210 (13)	−0.0031 (11)	−0.0045 (11)	0.0050 (11)
C30	0.0272 (14)	0.0183 (13)	0.0195 (12)	0.0023 (10)	0.0012 (10)	0.0028 (10)
C31	0.0222 (13)	0.0139 (12)	0.0259 (13)	0.0000 (10)	0.0001 (10)	0.0052 (10)
C32	0.0200 (12)	0.0182 (13)	0.0214 (12)	−0.0008 (10)	−0.0040 (10)	0.0035 (10)
O1W	0.0504 (15)	0.0448 (14)	0.0284 (11)	0.0117 (11)	0.0058 (10)	0.0067 (10)
O2W	0.0409 (13)	0.0325 (12)	0.0357 (12)	−0.0035 (10)	0.0007 (10)	0.0013 (9)
O3W	0.0317 (12)	0.0357 (12)	0.0321 (11)	−0.0066 (9)	−0.0024 (9)	0.0036 (10)

### Geometric parameters (Å, °)

**Table:**

O1—C1	1.370 (3)	C19—H19A	0.97
O1—C20	1.429 (3)	C19—H19B	0.97
O2—C6	1.371 (3)	C20—H20A	0.97
O2—C7	1.431 (3)	C20—H20B	0.97
O3—C8	1.418 (3)	O7—C21	1.323 (3)
O3—C9	1.434 (3)	O7—H1O7	0.99
O4—C11	1.367 (3)	O8—N1	1.223 (3)
O4—C10	1.434 (3)	O9—N1	1.225 (3)
O5—C16	1.373 (3)	O10—N2	1.214 (3)
O5—C17	1.435 (3)	O11—N2	1.217 (3)
O6—C18	1.423 (3)	O12—N3	1.213 (3)
O6—C19	1.424 (3)	O13—N3	1.219 (3)
C1—C2	1.380 (4)	N1—C22	1.474 (4)
C1—C6	1.412 (4)	N2—C24	1.470 (4)
C2—C3	1.400 (4)	N3—C26	1.457 (4)
C2—H2A	0.93	C21—C22	1.405 (4)
C3—C4	1.381 (4)	C21—C26	1.413 (4)
C3—H3A	0.93	C22—C23	1.372 (4)
C4—C5	1.398 (4)	C23—C24	1.380 (4)
C4—H4A	0.93	C23—H23A	0.93
C5—C6	1.379 (4)	C24—C25	1.370 (4)
C5—H5A	0.93	C25—C26	1.389 (4)
C7—C8	1.499 (4)	C25—H25A	0.93
C7—H7A	0.97	O14—C27	1.317 (3)
C7—H7B	0.97	O14—H14B	0.78
C8—H8A	0.97	O15—N4	1.216 (4)
C8—H8B	0.97	O16—N4	1.225 (4)
C9—C10	1.499 (4)	O17—N5	1.228 (4)
C9—H9A	0.97	O18—N5	1.216 (3)
C9—H9B	0.97	O19—N6	1.221 (3)
C10—H10A	0.97	O20—N6	1.234 (3)
C10—H10B	0.97	N4—C28	1.480 (4)
C11—C12	1.392 (4)	N5—C30	1.462 (4)
C11—C16	1.400 (4)	N6—C32	1.465 (4)
C12—C13	1.401 (4)	C27—C32	1.407 (4)
C12—H12A	0.93	C27—C28	1.412 (4)
C13—C14	1.368 (4)	C28—C29	1.368 (4)
C13—H13A	0.93	C29—C30	1.383 (4)
C14—C15	1.388 (4)	C29—H29A	0.93
C14—H14A	0.93	C30—C31	1.376 (4)
C15—C16	1.388 (4)	C31—C32	1.385 (4)
C15—H15A	0.93	C31—H31A	0.93
C17—C18	1.498 (4)	O1W—H1W1	0.86
C17—H17A	0.97	O1W—H2W1	0.94
C17—H17B	0.97	O2W—H1W2	0.85
C18—H18A	0.97	O2W—H2W2	0.85
C18—H18B	0.97	O3W—H1W3	0.85
C19—C20	1.498 (4)	O3W—H2W3	0.95
			
C1—O1—C20	117.1 (2)	O6—C18—H18B	109.4
C6—O2—C7	116.1 (2)	C17—C18—H18B	109.4
C8—O3—C9	110.0 (2)	H18A—C18—H18B	108.0
C11—O4—C10	117.6 (2)	O6—C19—C20	110.5 (2)
C16—O5—C17	116.5 (2)	O6—C19—H19A	109.5
C18—O6—C19	109.43 (19)	C20—C19—H19A	109.5
O1—C1—C2	125.3 (2)	O6—C19—H19B	109.5
O1—C1—C6	114.6 (2)	C20—C19—H19B	109.5
C2—C1—C6	120.1 (2)	H19A—C19—H19B	108.1
C1—C2—C3	120.0 (2)	O1—C20—C19	107.4 (2)
C1—C2—H2A	120.0	O1—C20—H20A	110.2
C3—C2—H2A	120.0	C19—C20—H20A	110.2
C4—C3—C2	120.0 (3)	O1—C20—H20B	110.2
C4—C3—H3A	120.0	C19—C20—H20B	110.2
C2—C3—H3A	120.0	H20A—C20—H20B	108.5
C3—C4—C5	120.1 (3)	C21—O7—H1O7	107.2
C3—C4—H4A	120.0	O8—N1—O9	125.1 (2)
C5—C4—H4A	120.0	O8—N1—C22	117.6 (2)
C6—C5—C4	120.3 (3)	O9—N1—C22	117.2 (2)
C6—C5—H5A	119.8	O10—N2—O11	124.3 (3)
C4—C5—H5A	119.8	O10—N2—C24	117.8 (2)
O2—C6—C5	125.4 (2)	O11—N2—C24	117.9 (2)
O2—C6—C1	115.1 (2)	O12—N3—O13	123.5 (2)
C5—C6—C1	119.5 (2)	O12—N3—C26	118.5 (2)
O2—C7—C8	108.3 (2)	O13—N3—C26	118.0 (2)
O2—C7—H7A	110.0	O7—C21—C22	117.2 (2)
C8—C7—H7A	110.0	O7—C21—C26	128.3 (2)
O2—C7—H7B	110.0	C22—C21—C26	114.5 (2)
C8—C7—H7B	110.0	C23—C22—C21	124.4 (2)
H7A—C7—H7B	108.4	C23—C22—N1	117.1 (2)
O3—C8—C7	110.6 (2)	C21—C22—N1	118.5 (2)
O3—C8—H8A	109.5	C22—C23—C24	117.6 (3)
C7—C8—H8A	109.5	C22—C23—H23A	121.2
O3—C8—H8B	109.5	C24—C23—H23A	121.2
C7—C8—H8B	109.5	C25—C24—C23	122.3 (3)
H8A—C8—H8B	108.1	C25—C24—N2	119.1 (2)
O3—C9—C10	109.6 (2)	C23—C24—N2	118.6 (2)
O3—C9—H9A	109.7	C24—C25—C26	118.4 (3)
C10—C9—H9A	109.7	C24—C25—H25A	120.8
O3—C9—H9B	109.7	C26—C25—H25A	120.8
C10—C9—H9B	109.7	C25—C26—C21	122.8 (2)
H9A—C9—H9B	108.2	C25—C26—N3	116.5 (2)
O4—C10—C9	107.1 (2)	C21—C26—N3	120.8 (2)
O4—C10—H10A	110.3	C27—O14—H14B	117.3
C9—C10—H10A	110.3	O15—N4—O16	124.6 (3)
O4—C10—H10B	110.3	O15—N4—C28	118.9 (3)
C9—C10—H10B	110.3	O16—N4—C28	116.5 (3)
H10A—C10—H10B	108.6	O18—N5—O17	124.1 (3)
O4—C11—C12	125.0 (3)	O18—N5—C30	117.9 (3)
O4—C11—C16	115.1 (2)	O17—N5—C30	118.0 (3)
C12—C11—C16	119.9 (2)	O19—N6—O20	123.3 (2)
C11—C12—C13	119.3 (3)	O19—N6—C32	118.6 (2)
C11—C12—H12A	120.3	O20—N6—C32	118.0 (2)
C13—C12—H12A	120.3	O14—C27—C32	126.7 (2)
C14—C13—C12	120.3 (3)	O14—C27—C28	118.7 (2)
C14—C13—H13A	119.8	C32—C27—C28	114.6 (2)
C12—C13—H13A	119.8	C29—C28—C27	123.2 (3)
C13—C14—C15	120.8 (3)	C29—C28—N4	116.8 (2)
C13—C14—H14A	119.6	C27—C28—N4	120.0 (2)
C15—C14—H14A	119.6	C28—C29—C30	119.0 (3)
C16—C15—C14	119.8 (3)	C28—C29—H29A	120.5
C16—C15—H15A	120.1	C30—C29—H29A	120.5
C14—C15—H15A	120.1	C31—C30—C29	121.5 (3)
O5—C16—C15	125.1 (3)	C31—C30—N5	119.0 (3)
O5—C16—C11	115.0 (2)	C29—C30—N5	119.5 (2)
C15—C16—C11	119.8 (2)	C30—C31—C32	118.0 (3)
O5—C17—C18	107.5 (2)	C30—C31—H31A	121.0
O5—C17—H17A	110.2	C32—C31—H31A	121.0
C18—C17—H17A	110.2	C31—C32—C27	123.6 (2)
O5—C17—H17B	110.2	C31—C32—N6	115.5 (2)
C18—C17—H17B	110.2	C27—C32—N6	120.9 (2)
H17A—C17—H17B	108.5	H1W1—O1W—H2W1	125.3
O6—C18—C17	111.0 (2)	H1W2—O2W—H2W2	107.7
O6—C18—H18A	109.4	H1W3—O3W—H2W3	115.7
C17—C18—H18A	109.4		
			
C20—O1—C1—C2	−9.1 (4)	O9—N1—C22—C21	−128.6 (3)
C20—O1—C1—C6	173.1 (2)	C21—C22—C23—C24	−3.3 (4)
O1—C1—C2—C3	−178.6 (2)	N1—C22—C23—C24	178.4 (2)
C6—C1—C2—C3	−1.0 (4)	C22—C23—C24—C25	1.4 (4)
C1—C2—C3—C4	0.1 (4)	C22—C23—C24—N2	−179.3 (2)
C2—C3—C4—C5	0.7 (4)	O10—N2—C24—C25	171.8 (3)
C3—C4—C5—C6	−0.6 (4)	O11—N2—C24—C25	−8.1 (4)
C7—O2—C6—C5	7.2 (4)	O10—N2—C24—C23	−7.6 (4)
C7—O2—C6—C1	−175.0 (2)	O11—N2—C24—C23	172.5 (3)
C4—C5—C6—O2	177.4 (3)	C23—C24—C25—C26	0.5 (4)
C4—C5—C6—C1	−0.3 (4)	N2—C24—C25—C26	−178.8 (2)
O1—C1—C6—O2	1.1 (3)	C24—C25—C26—C21	−0.7 (4)
C2—C1—C6—O2	−176.8 (2)	C24—C25—C26—N3	178.2 (3)
O1—C1—C6—C5	179.0 (2)	O7—C21—C26—C25	178.3 (3)
C2—C1—C6—C5	1.1 (4)	C22—C21—C26—C25	−0.9 (4)
C6—O2—C7—C8	−173.3 (2)	O7—C21—C26—N3	−0.6 (4)
C9—O3—C8—C7	174.3 (2)	C22—C21—C26—N3	−179.8 (2)
O2—C7—C8—O3	−73.2 (3)	O12—N3—C26—C25	5.1 (4)
C8—O3—C9—C10	179.1 (2)	O13—N3—C26—C25	−173.7 (3)
C11—O4—C10—C9	175.4 (2)	O12—N3—C26—C21	−175.9 (3)
O3—C9—C10—O4	71.5 (3)	O13—N3—C26—C21	5.3 (4)
C10—O4—C11—C12	−8.7 (4)	O14—C27—C28—C29	177.0 (3)
C10—O4—C11—C16	173.5 (2)	C32—C27—C28—C29	−2.1 (4)
O4—C11—C12—C13	−176.6 (3)	O14—C27—C28—N4	−4.0 (4)
C16—C11—C12—C13	1.1 (4)	C32—C27—C28—N4	176.8 (2)
C11—C12—C13—C14	−0.5 (4)	O15—N4—C28—C29	146.4 (3)
C12—C13—C14—C15	−0.3 (5)	O16—N4—C28—C29	−32.1 (4)
C13—C14—C15—C16	0.5 (5)	O15—N4—C28—C27	−32.6 (4)
C17—O5—C16—C15	−2.1 (4)	O16—N4—C28—C27	148.9 (3)
C17—O5—C16—C11	175.8 (2)	C27—C28—C29—C30	2.6 (4)
C14—C15—C16—O5	177.9 (3)	N4—C28—C29—C30	−176.4 (2)
C14—C15—C16—C11	0.1 (4)	C28—C29—C30—C31	−1.1 (4)
O4—C11—C16—O5	−1.0 (3)	C28—C29—C30—N5	−179.7 (2)
C12—C11—C16—O5	−178.9 (2)	O18—N5—C30—C31	4.1 (4)
O4—C11—C16—C15	177.0 (2)	O17—N5—C30—C31	−175.3 (3)
C12—C11—C16—C15	−0.9 (4)	O18—N5—C30—C29	−177.2 (3)
C16—O5—C17—C18	−166.7 (2)	O17—N5—C30—C29	3.4 (4)
C19—O6—C18—C17	176.9 (2)	C29—C30—C31—C32	−0.7 (4)
O5—C17—C18—O6	−69.8 (3)	N5—C30—C31—C32	177.9 (2)
C18—O6—C19—C20	−177.3 (2)	C30—C31—C32—C27	1.2 (4)
C1—O1—C20—C19	179.0 (2)	C30—C31—C32—N6	178.9 (2)
O6—C19—C20—O1	72.0 (3)	O14—C27—C32—C31	−178.9 (3)
O7—C21—C22—C23	−176.3 (3)	C28—C27—C32—C31	0.2 (4)
C26—C21—C22—C23	3.0 (4)	O14—C27—C32—N6	3.6 (4)
O7—C21—C22—N1	2.0 (4)	C28—C27—C32—N6	−177.4 (2)
C26—C21—C22—N1	−178.7 (2)	O19—N6—C32—C31	0.9 (4)
O8—N1—C22—C23	−128.7 (3)	O20—N6—C32—C31	−178.9 (2)
O9—N1—C22—C23	49.9 (4)	O19—N6—C32—C27	178.6 (2)
O8—N1—C22—C21	52.9 (4)	O20—N6—C32—C27	−1.2 (4)

### Hydrogen-bond geometry (Å, °)

**Table:**

*D*—H···*A*	*D*—H	H···*A*	*D*···*A*	*D*—H···*A*
O7—H1O7···O3W	1.00	1.92	2.618 (3)	124
O7—H1O7···O13	1.00	1.84	2.664 (3)	138
O7—H1O7···N3	1.00	2.49	2.985 (3)	110
O1W—H1W1···O6	0.87	2.14	2.978 (3)	162
O1W—H2W1···O2W	0.94	2.03	2.900 (4)	152
O2W—H1W2···O1^i^	0.85	2.56	3.215 (3)	135
O2W—H1W2···O2^i^	0.85	2.45	3.265 (3)	162
O2W—H2W2···O4^i^	0.85	2.42	3.198 (3)	152
O2W—H2W2···O5^i^	0.85	2.43	3.155 (3)	144
O3W—H1W3···O3	0.85	2.02	2.861 (3)	173
O3W—H2W3···O1W	0.95	1.96	2.881 (3)	163
O14—H14B···O1W	0.78	2.06	2.732 (3)	144
O14—H14B···O20	0.78	2.05	2.632 (3)	131
C3—H3A···O11^ii^	0.93	2.60	3.323 (4)	136
C7—H7A···O6^iii^	0.97	2.58	3.393 (3)	142
C7—H7B···O19^iv^	0.97	2.59	3.135 (4)	116
C9—H9A···O12^iii^	0.97	2.39	3.341 (4)	165
C19—H19A···O19^v^	0.97	2.53	3.301 (3)	137

Symmetry codes:  (i) *x*, *y*+1, *z*; (ii) −*x*+1/2, *y*−1/2, *z*+1/2; (iii) *x*−1/2, −*y*+1/2, *z*; (iv) *x*, *y*−1, *z*; (v) *x*+1/2, −*y*+1/2, *z*.
